# Gender Difference in Symptom Presentations Among Patients With Bone Metastases in Gender-Specific and Gender-Neutral Primary Cancers

**DOI:** 10.4021/wjon306w

**Published:** 2011-06-08

**Authors:** Shaelyn Culleton, Kristopher Dennis, Kaitlin Koo, Liying Zhang, Liang Zeng, Janet Nguyen, Florencia Jon, Lori Holden, Elizabeth Barnes, May Tsao, Cyril Danjoux, Arjun Sahgal, Edward Chow

**Affiliations:** a Rapid Response Radiotherapy Program, Department of Radiation Oncology, Odette Cancer Center, Sunnybrook Health Sciences Center, University of Toronto, Canada

**Keywords:** Advanced cancer, BPI, ESAS, Gender difference, Symptoms

## Abstract

**Background:**

Studies have assessed gender differences on symptoms commonly experienced by cancer patients at various stages in their disease trajectory using heterogeneous cancer populations with different tumor types. The purpose of our study was to evaluate the effect of gender on symptoms among patients with bone metastases while controlling for gender-specific malignancies.

**Methods:**

A retrospective review of patients receiving palliative radiotherapy for bone metastases was conducted on patients that completed the Brief Pain Inventory (BPI) or Edmonton Symptom Assessment System (ESAS) questionnaires from 1999 - 2004. Baseline and follow-up BPI and ESAS symptom scores were compared between males and females, with and without controlling for gender-specific tumors.

**Results:**

A total of 900 patients completed baseline questionnaires: ESAS (n = 508) or BPI (n = 392). The most common tumor types were lung (26%), breast (25%) and prostate (24%). In all ESAS patients, females had significantly greater severity of tiredness, nausea, depression, anxiety and breathlessness. In the subgroup analysis when gender-specific primary cancers were removed (i.e., breast, prostate and gynecological), no significant differences in ESAS symptoms were found between genders. The BPI functional item of walking ability was significantly worse for females in both the overall and subgroup analyses. Females had worse symptoms at follow-up prior to the removal of gender-specific primaries in both ESAS and BPI.

**Conclusions:**

Gender-specific cancers may significantly bias gender studies of cancer-related symptoms when primary tumor type is not taken into account. Gender differences are best assessed in gender-neutral primaries.

## Introduction

Patients with advanced cancer often experience a variety of physical, functional and psychosocial symptoms in association with their disease, which in turn can have a significant impact on their quality of life (QoL) [1]. These patients are often reported to be ‘polysymptomatic’ with respect to their advanced cancer [2, 3]. In addition to physical symptoms, the incidence of psychological disturbances has been estimated from 0 - 49% in the general cancer population, however significant levels of depression and anxiety have only been found to be around 19% and 24% respectively [4, 5]. In an effort to correlate possible demographic and treatment-related variables to symptoms commonly associated with cancer, many of these studies have found that younger patients [1, 5-11], patients with poorer performance status [11-13] and females [1, 5, 9-12, 14, 15] are more likely to experience psychological distress and/or symptoms of anxiety and depression. Female cancer patients have also been reported to experience more nausea and/or vomiting as well as more nociceptive pain when compared with male cancer patients [2, 3, 10, 11, 16-18].

Most of the above studies used heterogeneous cancer populations that included various tumor types and disease stages. Specifically with respect to gender differences, many meta-analyses and studies with more uniform populations of cancer patients have found no concrete evidence in support of such reported gender related symptom differences [6, 19-24]. Very few studies have acknowledged the potential bias in including breast, prostate and gynecological cancer sites in correlational studies, particularly when they comment on gender related differences in the prevalence and severity of symptoms. Some authors have noted that gender-specific primary sites may influence the pattern and severity of symptoms and that these differences may be attributed to different hormone and chemotherapy treatments, the public’s perception of specific cancers as well as different self-reporting styles between the sexes [1, 3, 10, 16, 25-27]. It has been suggested that in order to elucidate the true effect of gender on symptoms in the cancer population, future studies should evaluate the gender effect in patients with similar distributions of primary cancers or in patients with cancers that are not gender-specific [1, 28]. Accordingly, the purpose of this study was to evaluate the effect that gender-specific primaries may have on commonly reported physical, functional and psychosocial symptoms in a population of advanced cancer patients with bone metastases.

## Methods

### Patient population

A retrospective analysis was conducted on data from patients who had participated in only one of two previous mutually exclusive prospective longitudinal studies that occurred between 1999 and 2004. One used the Edmonton Symptom Assessment System (ESAS) and the other used the Brief Pain Inventory (BPI). Both studies assessed patients at baseline and at post-treatment follow-up. Patients were to be assessed at follow-up at 1, 2, 4 weeks after the radiation treatment and then monthly afterwards, and we allowed a window phase of 1 week during these designated times of follow-up. Patients were accrued from an outpatient radiotherapy clinic that operated daily at the Sunnybrook Odette Cancer Center, Toronto, Ontario, Canada. Ethics approval was received for this study from the hospital’s research ethics board. Patients considered for enrolment in the aforementioned studies had to have been over 18 years of age, had biopsy-proven cancer, spoke English, and received palliative radiotherapy (RT) for painful bone metastases.

### Edmonton Symptom Assessment System (ESAS)

The ESAS is a validated 9-item patient-rated tool where symptoms are scored on an 11-point visual analogue scale where 0 is absence of a symptom and 10 is the worst possible feeling or experience of that symptom. The symptoms assessed by the ESAS are pain, tiredness, nausea, depression, anxiety, drowsiness, appetite loss, well-being and breathlessness. The tool was initially developed for the palliative care setting and is known for its brevity, reliability and ease of completion [29, 30].

### Brief Pain Inventory (BPI)

The BPI is a validated inventory that assesses the severity and impact of pain. It asks patients to rate their current, average and worst pain on an 11-point visual analogue scale where 0 is ‘no pain’ and 10 is ‘pain as bad as you can imagine’. Patients are also asked to rate how much pain interferes with their enjoyment of life, level of activity, ability to walk, mood, sleep, work and relations with others on a 11-point visual analogue scale where 0 is defined as ‘does not interfere’ and 10 is defined as ‘completely interferes’. This tool has been utilized in patients with bone metastases and is known for its reliability and sensitivity [31].

### Statistical analysis

Patients enrolled on both the ESAS and BPI studies who completed the ESAS and BPI were analysed in two separate groups: overall and subgroup patients. The overall analyses of the ESAS and BPI groups respectively were conducted in all patients including all primary cancer sites. The separate subgroup analyses excluding all gender-specific primaries (breast, prostate and gynecological cancers) were repeated. Demographic results were expressed as median and range for age and KPS; as proportions for primary cancer site in overall patients and in males and females, respectively. Because both ESAS and BPI score distributions were not normalized, Wilcoxon rank-sum non-parametric tests were used to compare males and females for each ESAS and BPI score at baseline and at each follow-up. A P-value of less than 0.05 was considered statistically significant. All analyses were conducted by Statistical Analysis Software (SAS version 9.2 for Windows).

## Results

In total, 900 patients were included in the present analysis. Five hundred and eight had been previously enrolled on the ESAS study and 392 had been previously enrolled on the BPI study. During 2004 - 2009 (the period during which patients for the current analysis was enrolled to these studies), 3350 patients were referred to the outpatient clinic from which all patients were enrolled. During a typical year, 60% of all new referrals to the clinic are patients with painful bone metastases. Detailed information about patients not enrolled to studies is not retained.

### Edmonton Symptom Assessment System

#### All patients

A total of 508 patients completed the ESAS at baseline prior to palliative RT. Their median age was 69 years (range 32 - 94) and their median Karnofsky performance status (KPS) was 60 (range 10 - 100). The three most common primary cancer sites were lung (25%), breast (25%) and prostate (23%); and there were slightly more males (54%) than females (46%). All other demographic information is reported in Table 1. When assessing baseline ESAS scores in all patients, the mean score of the three worst items were tiredness (4.98), pain (4.36) and well-being (4.35). When evaluating gender differences in all ESAS items, tiredness (P = 0.0422), nausea (P = 0.0249), depression (P = 0.0394), anxiety (P = 0.0485) and breathlessness (P = 0.0135) were significantly worse in females than males. All other baseline ESAS scores can be found in Table 2.

**Table 1:** Baseline ESAS Cohort Demographics

	All Patients			Sub-Analysis		
	M	F	Overall	M	F	Overall
N	275	233	508	158	104	262
%	54%	46%	100%	60%	40%	100%
Age						
Median (years)	71	67	69	67	69	68
Range	37 - 94	59 - 76	32 - 94	37 - 86	32 - 89	61 - 75
KPS						
Median	60	60	60	60	60	60
Range	10 - 90	30 - 100	10 - 100	40 - 90	30 - 90	30 - 90
Primary Cancer Site (N =; %)						
Lung	78 (28%)	50 (21%)	128 (25%)	78 (49%)	50 (48%)	128 (49%)
Breast	---	125 (54%)	125 (25%)	---	---	---
Prostate	117 (43%)	---	117 (23%)	---	---	---
Unknown	17 (6%)	16 (7%)	66 (7%)	17 (11%)	16 (15%)	33 (13%)
Multiple Myeloma	15 (5%)	13 (6%)	28 (6%)	15 (10%)	13 (13%)	28 (11%)
Colorectal	18 (7%)	9 (4%)	27 (5%)	18 (11%)	9 (9%)	27 (10%)
Renal Cell	10 (4%)	5 (2%)	15 (3%)	10 (7%)	5 (5%)	15 (6%)
Other GI	10 (4%)	1 (1%)	11 (2%)	10 (7%)	1 (1%)	11 (4%)
Bladder	6 (2%)	3 (1%)	9 (2%)	6 (4%)	3 (3%)	9 (3%)
GYN	---	4 (2%)	4 (1%)	---	---	---
Others	4 (1%)	7 (3%)	11 (2%)	4 (3%)	7 (7%)	11 (4%)
Dose/Fraction (N =; %)						
800/1	127 (46%)	91 (39%)	218 (43%)	71 (45%)	38 (37%)	109 (42%)
2000/5	108 (39%)	105 (45%)	213 (42%)	62 (39%)	45 (43%)	107 (41%)
3000/10	13 (5%)	13 (6%)	26 (5%)	8 (5%)	7 (7%)	15 (6%)
Other	27 (10%)	24 (10%)	51 (10%)	17 (11%)	14 (13%)	31 (12%)

GI: Gastrointestinal; GYN: Gynecological

**Table 2:** Baseline ESAS Symptoms and Scores

ESAS symptom	All Patients			Sub-Analysis		
	Mean Score		P-value	Mean Score		P-value
	Male	Female		Male	Female	
Pain	4.3	4.4	0.68	4.7	4.1	0.12
Tiredness	4.7	5.3	0.0422*	4.9	5.5	0.15
Nausea	1.3	1.8	0.0249*	1.5	1.4	0.81
Depression	2.3	2.8	0.0394*	2.5	2.8	0.25
Anxiety	3.0	3.6	0.0485*	3.2	3.5	0.47
Drowsiness	3.5	3.7	0.35	3.6	4.3	0.07
Appetite Loss	3.9	4.4	0.10	4.2	4.7	0.26
Well-Being	4.2	4.6	0.10	4.2	4.6	0.30
Breathlessness	1.9	2.6	0.0153*	2.2	2.5	0.69

* Females were significantly worse than males.

At follow-up, all items with the exception of dyspnoea were significantly worse for females at some point during the follow-up period. The two most frequently occurring items that showed a significant worsening for females during follow-up were appetite loss at weeks 2, 11, and 12 and nausea at weeks 2, 4, and 6. At no point did males experience worse ESAS symptoms throughout the follow-up period. All other follow-up data is reported in Table 3.

**Table 3:** Comparison Male and Female ESAS Item Results During Follow-up After Palliative RT for Bone Metastases

Weeks		1	2	3	4	5	6	7	8	9	10	11	12	13	14	15	16
All Patients																	
N	Male	88	109	64	92	55	23	11	47	42	24	19	43	28	19	10	7
	Female	71	94	82	63	59	28	21	52	43	27	16	45	45	17	11	5
Pain				*		*											
Tiredness			*														
Nausea			*		*		*										
Depression										*		*					
Anxiety															*		
Drowsiness			*							*							
Appetite Loss			*									*	*				
Well-Being										*							
Dyspnoea																	
Sub-Analysis																	
N	Male	43	59	42	47	23	13	8	22	24	13	13	27	12	10	7	1
	Female	29	33	29	27	18	8	6	18	17	5	7	12	13	7	4	3
Pain				*													
Tiredness			*											*			
Nausea																	
Depression																	
Anxiety															*		
Drowsiness		*	*														
Appetite Loss												*					
Well-Being										*							
Dyspnoea																	

* Statistically significant difference between genders (P < 0.05)

#### Sub-analysis excluding patients with gender-specific primaries

A total of 262 patients were included in the subgroup analysis of gender-neutral primaries. The median age was 68 years (range 32 - 89) and the median KPS was 60 (30 - 90). Again there were slightly more males (60%) than females (40%) but both genders shared very similar demographic information. The most common primary cancer sites were lung (49%), unknown (13%), multiple myeloma (11%) and colorectal (10%). All other demographic information can be found in Table 1. When assessing baseline ESAS score within this sub-group, the mean score of the three worst items were tiredness (5.11), pain (4.42) and appetite loss (4.38). When evaluating gender differences, there were no significant differences in all ESAS scores at baseline. All other baseline ESAS scores can be found in Table 2.

At follow-up, all items with the exception of nausea, depression and breathlessness were significantly worse in females at some point during the follow-up period. However, as seen in Table 3, the number of items that were significantly worse in females during follow-up dropped to 8 from 15 when compared to the analysis that included all patients. The two most frequently occurring items that showed a significant worsening for females during the follow-up were tiredness at weeks 2 and 13, and drowsiness at weeks 1 and 2. At no point did males have significantly worse scores than females throughout the follow-up period.

### Brief Pain Inventory

#### All patients

A total of 392 patients completed the BPI at baseline. Their median age was 68 years (range 30 - 91) and the median KPS was 70 (range 30 - 90). There were more males (60%) than females (40%), however the demographic variables were comparable between both genders at baseline. The most common primary cancer sites were lung (27%), breast (25%) and prostate (25%). All other demographic information is displayed in Table 4. The three items that pain interfered with the most were normal work (6.86), general activity (6.66) and overall enjoyment of life (6.60). When analyzed by gender, the only BPI item that was significantly worse in females was walking ability (P = 0.0247). All other BPI items showed no significant difference between genders. Additional baseline BPI information is presented in Table 5.

**Table 4:** Baseline BPI Cohort Demographics

	All Patients			Sub-Analysis		
	M	F	Overall	M	F	Overall
N	227	165	392	126	67	193
%	58%	42%	100%	65%	35%	100%
Age						
Median (years)	70	61	68	67	66	67
Range	30 - 91	31 - 89	30 - 91	33 - 86	40 - 85	33 - 86
KPS						
Median	70	70	70	70	70	70
Range	30 - 90	30 - 90	30 - 90	40 - 90	40 - 90	40 - 90
Primary Cancer Site (N; %)						
Lung	68 (30%)	36 (22%)	104 (27%)	68 (54%)	36 (54%)	104 (54%)
Breast	2 (1%)	96 (58%)	98 (25%)	---	---	---
Prostate	98 (43%)	---	98 (25%)	---	---	---
Renal Cell	17 (7%)	5 (3%)	22 (6%)	17 (14%)	5 (8%)	22 (11%)
Colorectal	15 (7%)	7 (4%)	22 (6%)	15 (12%)	7 (10%)	22 (11%)
Unknown	9 (4%)	9 (6%)	18 (5%)	9 (7%)	9 (13%)	18 (9%)
Bladder	7 (3%)	5 (3%)	12 (3%)	7 (6%)	5 (7%)	12 (6%)
Other GI	5 (2%)	3 (2%)	8 (2%)	5 (4%)	3 (4%)	8 (4%)
GYN	---	2 (1%)	2 (1%)	---	---	---
Others	6 (3%)	2 (1%)	8 (2%)	5 (4%)	2 (3%)	7 (4%)

GI: Gastrointestinal; GYN: Gynecological

**Table 5:** Baseline BPI Items and Scores

BPI Items	All Patients			Sub-Analysis		
	Mean Score		P-value	Mean Score		P-value
	Male	Female		Male	Female	
Worst Pain	7.1	7.8	No data	7.2	8.2	No data
Average Pain	4.9	5.2	No data	5.0	5.3	No data
Current Pain	3.5	4.0	No data	4.0	4.5	No data
General Activity	6.5	6.9	0.27	6.7	7.6	0.10
Mood	4.9	5.4	0.16	5.0	5.6	0.31
Walking Ability	6.1	6.3	0.0247*	5.6	7.0	0.0075*
Normal Work	6.7	7.1	0.52	7.0	8.0	0.34
Relationship	3.2	3.7	0.22	3.9	4.2	0.65
Sleeping	4.8	4.9	0.78	4.8	5.7	0.14
Enjoyment of Life	6.6	6.6	0.81	6.7	7.3	0.27

* Females were significantly worse than males.

At follow-up, all BPI items were significantly worse in females at some point during the follow-up with the exception of mood. At no point during the follow-up did males experience worse symptoms than females. The most common items that pain had significantly interfered with at follow-up in females were relationships with other people at weeks 4, 8, and 10; general activity at weeks 5 and 9; normal work at weeks 4 and 10; and sleep problems at weeks 8 and 10. All other follow-up information can be found in Table 6.

**Table 6:** BPI Follow-up Results After Palliative RT for Bone Metastases

Weeks		3	4	5	6	7	8	9	10	11	12	13	14	15	16
All Patients															
N	Male	41	79	24	14	21	51	34	12	16	39	32	15	17	17
	Female	23	61	27	6	19	35	20	6	23	20	13	11	13	15
General Activity				*				*							
Mood															
Walking Ability				*											
Normal Work			*						*						
Relationship			*				*		*						
Sleeping							*		*						
Enjoyment of Life								*							
Sub-Analysis															
N	Male	20	37	16	5	10	17	21	6	5	15	19	7	4	7
	Female	11	24	12	1	7	15	9	1	6	7	8	4	3	6
General Activity															
Mood															
Walking Ability				*											
Normal Work							*								
Relationship							*								
Sleeping							*								
Enjoyment of Life															

* Statistically significant difference between genders (P < 0.05)

#### Sub-analysis excluding gender-specific cancers

A total of 191 patients were included in the sub-analysis of patients with non gender-specific primaries. The median age was 67 years (range 57 - 74) and the median KPS was 70 (range 40 - 90). There were more males (65%) than females (35%), however demographic variables were comparable in both groups. The most common cancer sites were lung (54%), renal cell (11%) and colorectal (11%). All other demographic variables are presented in Table 4. The three items that pain interfered with the most were normal work (7.35), general activity (7.02) and enjoyment of life (6.95). When analyzed by gender, the same baseline BPI item found in all patients (walking ability) was still significantly worse in females (P = 0.0075). All other BPI items showed no significant difference between genders. All other baseline BPI information can be found in Table 5.

At follow-up, several of the associations in the overall analysis between BPI items and females were no longer significant. The only BPI items that were significantly worse in females were normal work, relationships with others and sleep problems at week 8, and walking ability at week 5. The number of significant items dropped to 4 from 11 when compared to all patients that were analyzed. All other follow-up information can be found in Table 6.

## Discussion

Despite several studies that have assessed gender’s influence on the prevalence and severity of common cancer symptoms, there is still no consensus regarding the role and extent gender may play [3, 32]. Patients with cancer can experience similar levels of anxiety and depression as the general population and epidemiological studies have consistently reported a higher incidence of depression and anxiety among females [33, 34]. Reviews and meta-analyses of the published literature however, have reported that the results of studies that have analyzed depression and anxiety between genders in cancer patients are confounded by differences in age, tumor type and prognosis; and that these gender differences may not be seen in this population [4, 23, 35]. Certain tumor types for example, have long been linked to higher frequencies of depression and anxiety. With respect to depression, these include pancreatic, head and neck, breast and lung cancers [4, 5, 9, 36-40]. Tumor types that have been associated with increased prevalence of anxiety include pancreatic, thyroid, breast and gynecological cancers [1, 7, 41]. Some authors have postulated that pathophysiological effects of these tumors, particularly in their differential expression of enzymes, neurotransmitters, hormones and cytokines could create paraneoplastic syndromes that elevate levels of depression and anxiety [3, 5, 38, 39]. Different chemotherapy and hormone therapy treatments, such as tamoxifen, as well as the use of steroids have also been linked to elevated levels of depression and anxiety in cancer patients [6, 10, 25, 37, 39, 40, 42, 43]. Therefore tumor type and their respective treatments may play an important role in the severity and prevalence of anxiety and depression; and the inclusion of gender-specific tumors, such as breast cancer, may artificially increase the perceived rates of anxiety and/or depression among females in the cancer populationADDIN RW.CITE{{195 Parker,P.A. 2003}}. Our study findings certainly demonstrated this when upon exclusion of gender-specific primaries, the severity of anxiety and depression were no longer found to be significantly greater in females. Conversely, prostate cancer patients have often been reported to experience lower rates of depression and anxiety; and men in general have been found to under-report their symptoms [1, 9, 27, 44]. This could have also influenced the results of the sub-analysis. Other factors that may partially explain our results are recent findings that suggest the gender gap with respect to depression in the general population appears to be narrowing as gender roles become more similar and less traditional; and that perceived gender differences may not appear in the cancer population because the stressors and emotional burden that accompany the disease possibly negate these differences [21, 25, 33].

In addition to the anxiety and depression, the other symptoms that were evaluated by the ESAS and were found not to vary as a function of gender after the sub-analysis were nausea, tiredness, and dyspnoea. Less research had been devoted to the gender related differences of these symptoms and the majority of such studies have analyzed this relationship in large heterogeneous cancer populations where only the prevalence of these symptoms has been captured. With respect to nausea, two large studies with over 1500 patients each with various tumor types and stages found a high prevalence of nausea in the female population. However both of these studies included gender-specific primaries and upon analysis of symptoms by cancer site, a high prevalence of nausea was found in gynecological and breast cancer patients [17, 18]ADDIN RW.CITE{{237 Cheung,W. 2010}}. Both of these studies commented that the significantly greater prevalence of nausea in women could very well be attributed to their primary cancer sites or the highly emetogenic chemotherapy regimens [10, 17, 18].

In the advanced cancer setting, four studies with patient populations ranging from 325 - 1358 patients each evaluated nausea and also found it was significantly greater in females [2, 3, 10, 16]. However three of the studies found that with analysis of nausea and cancer site, gastrointestinal and breast cancers were significantly associated with patients’ experience of nausea and therefore this could have had an impact on the gender findings [3, 10, 16]. Only the study by Walsh et al. [2] still observed a gender difference in females’ experience of nausea when controlling for gender-specific primaries, however this study only collected the prevalence of specific symptoms rather than capturing severity or frequency.

Apart from nausea, tiredness or fatigue is one of the most frequent and persistent symptoms of cancer and in the advanced cancer setting it has been reported to be present in as many as 89% of patients [24, 45]. It has been correlated with high levels of psychological morbidity [45, 46] in young and early stage cancer patients, but has been shown to be better correlated with worsening physical symptoms such as pain and dyspnoea in the advanced cancer setting [24]. In a review by Miaskowski et al. [42] that analyzed fatigue in cancer patients, studies that reported greater severity in female patients once again used heterogeneous populations in their analyses. In two unpublished studies by Miaskowski et al. [42], one of which analyzed gender differences in fatigue in 198 advanced cancer patients with bone metastases; as well as in two other studies of 95 and 1358 advanced cancer patients respectively, no gender differences were found [10, 24]. In addition, several studies that have only analyzed the lung cancer population have also reported no gender differences with respect to fatigue [19, 42, 47]. Of interest, one study published by Engel and colleagues [40] evaluated fatigue as well as other QoL parameters in 988 breast cancer patients and compared them to 327 rectal cancer patients. They found the breast cancer patients reported significantly higher rates of fatigue when compared to both the female and male rectal cancer patients, suggesting that breast cancer patients may artificially inflate gender related differences. This same study also reported that breast cancer patients, particularly those treated with adjuvant therapy, reported more dyspnoea than the rectal cancer patients.

Dyspnoea is reportedly present in about 11 - 28% of cancer patients and as expected it is found significantly more often in lung cancer patients [13, 16-18, 48]. Very few studies have included dyspnoea as a variable in gender related studies, however of those that did, no significant differences have been reported between genders [2, 10, 13, 16, 18].

With regards to the patients that had completed the BPI, one functional symptom was significant in both the overall and the non-gender-specific sub-analysis. Females reported that pain had more interference on their walking ability than males, despite both groups reporting similar current, average and worst pain scores. Most studies do not report a gender difference with respect to functional symptoms; however none of these studies have evaluated this outcome in an advanced cancer population of patients with bone metastases [17, 25, 28]. Patients with painful sites in the lower extremities requiring radiation treatment have been reported to experience greater functional inference [49]; however there was no difference between the genders regarding lower extremity treatment sites to explain the phenomenon. After palliative RT treatment, the only other time females experienced significantly more interference in their walking ability was at week 5 and it was not seen beyond this time point.

Briefly, with respect to follow-ups in both the ESAS and BPI patients, the number of significant gender differences upon exclusion of gender-specific primaries dropped by 47% and 64% respectively. There were no symptoms or functional interference items that are consistently worse among females in the sub-analysis. The greatest number of follow-ups for ESAS was at week 2 (40% of total accrued) and at week 4 for BPI patients (36% of total accrued). Therefore the interpretation of any significant results with respect to gender differences, especially beyond week 4, may not be representative or particularly meaningful. The only symptoms which were significantly greater in females at more than one time period were tiredness and drowsiness at weeks 1, 2, and 13.

Existing studies that have evaluated gender differences in heterogeneous cancer populations that include breast, prostate and gynecological cancers should be appraised critically, especially when they have not accessed the effects tumor type, cancer stage or other demographic factors may have on the severity and frequency of symptoms in the population. A clear-cut example of the potential influence gender-specific primaries may have on study outcomes can also be seen in the study by Engel et al. [40]. In a comparison of breast and rectal cancer patients, breast cancer patients were found to have significantly poorer scores on over half of the QoL items, including emotional functioning, fatigue, pain and insomnia even when controlling for adjuvant treatment, gender and age. The authors reported that this might be explained by the public’s perception of breast cancer and women’s fear of developing the disease due to the heightened publicity it receives when compared to other cancers. Since breast cancer has the highest reported incidence of female cancers [50], it can easily impact gender studies and artificially inflate the severity and prevalence of symptoms in females. Therefore one should not assume that symptom severity and frequency in patients with gender-specific primaries are generalizable to the entire cancer population.

The limitations of this study are primarily centered on the type of cancer population assessed. In order to keep a more homogenous population, the 900 patients all had bone metastases and received palliative RT to their painful bony sites. The results of this study therefore may not be representative of patients with early-stage cancer or patients with other metastases. In addition, other limitations of this study include those methodological concerns that are typically encountered in studies of patients with advanced cancer. There was a high attrition rate presumably due to patients suffering from declining performance status, competing health concerns or death. There was likely also greater relative accrual of patients with good performance status than patients with advanced cancer in general. Finally, although the size of this exploratory study is large at 900 patients, given the similarly high number of statistical comparisons needed to properly explore the main study question, more rigorous corrections for multiple comparisons were not performed as they could have easily masked the hypothesis generating findings found herein despite the large study cohort.

Overall, this study clearly demonstrates the importance of controlling for the influence of gender-specific primaries when investigating the effect of gender on the prevalence and severity of symptoms among patients with advanced cancer. Future studies should always take into account gender-specific primaries through multivariate analyses or by sub-analyses when analyzing gender relationships in the cancer population.
